# Supplementary figures and images for: Simultaneous cyclin D1 overexpression and p27kip1 knockdown enable robust Müller glia cell cycle reactivation in uninjured mouse retina
Source: eLife. 2025 Apr 3;13:RP100904. doi: 10.7554/eLife.100904 (PMC11968108; doi:10.7554/eLife.100904)

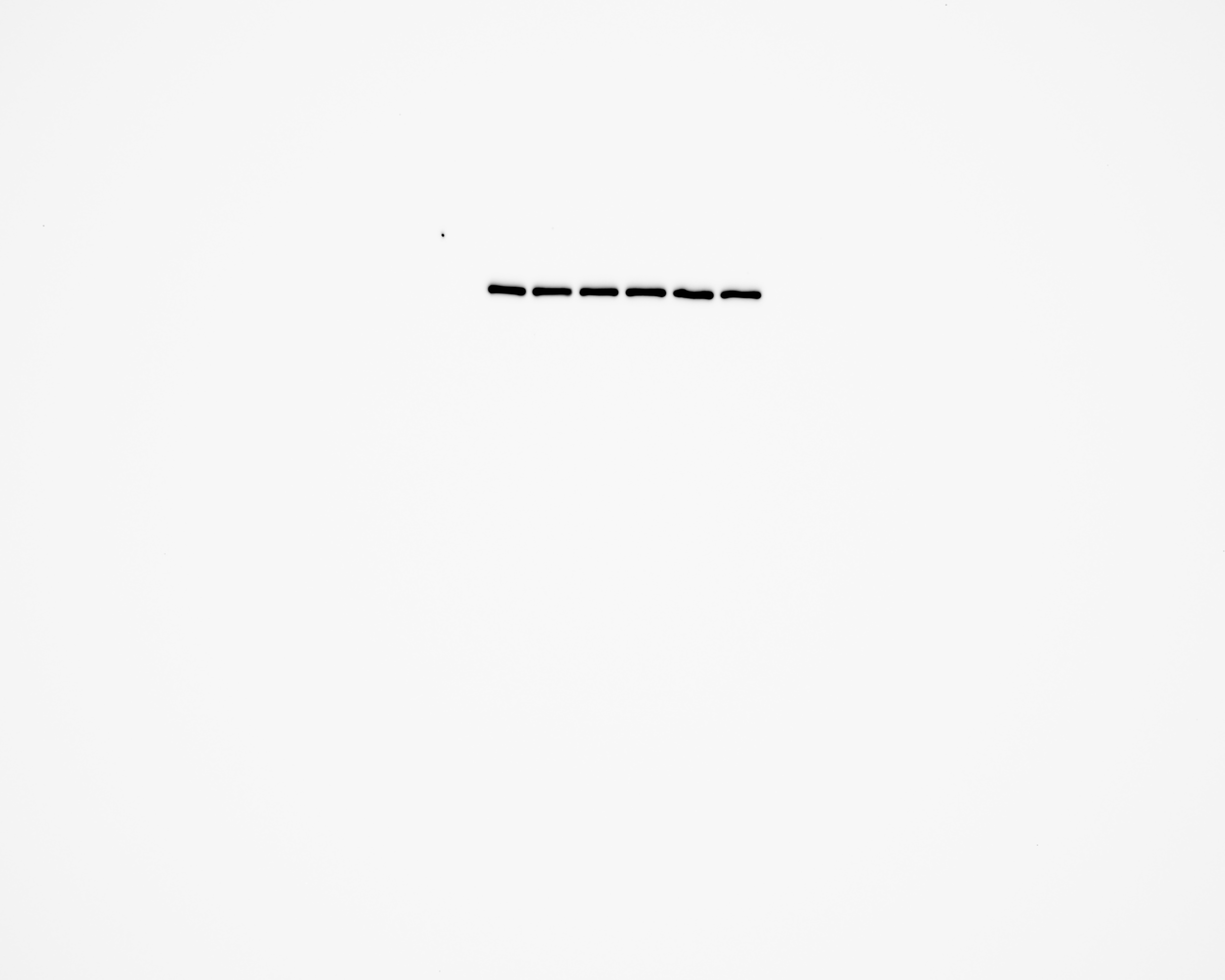

Supplement: Figure 1—figure supplement 3—source data 1. [file elife-100904-fig1-figsupp3-data1.zip › Supplementary Figure 3-source data /Anti-╬▒ tubulin.tif]

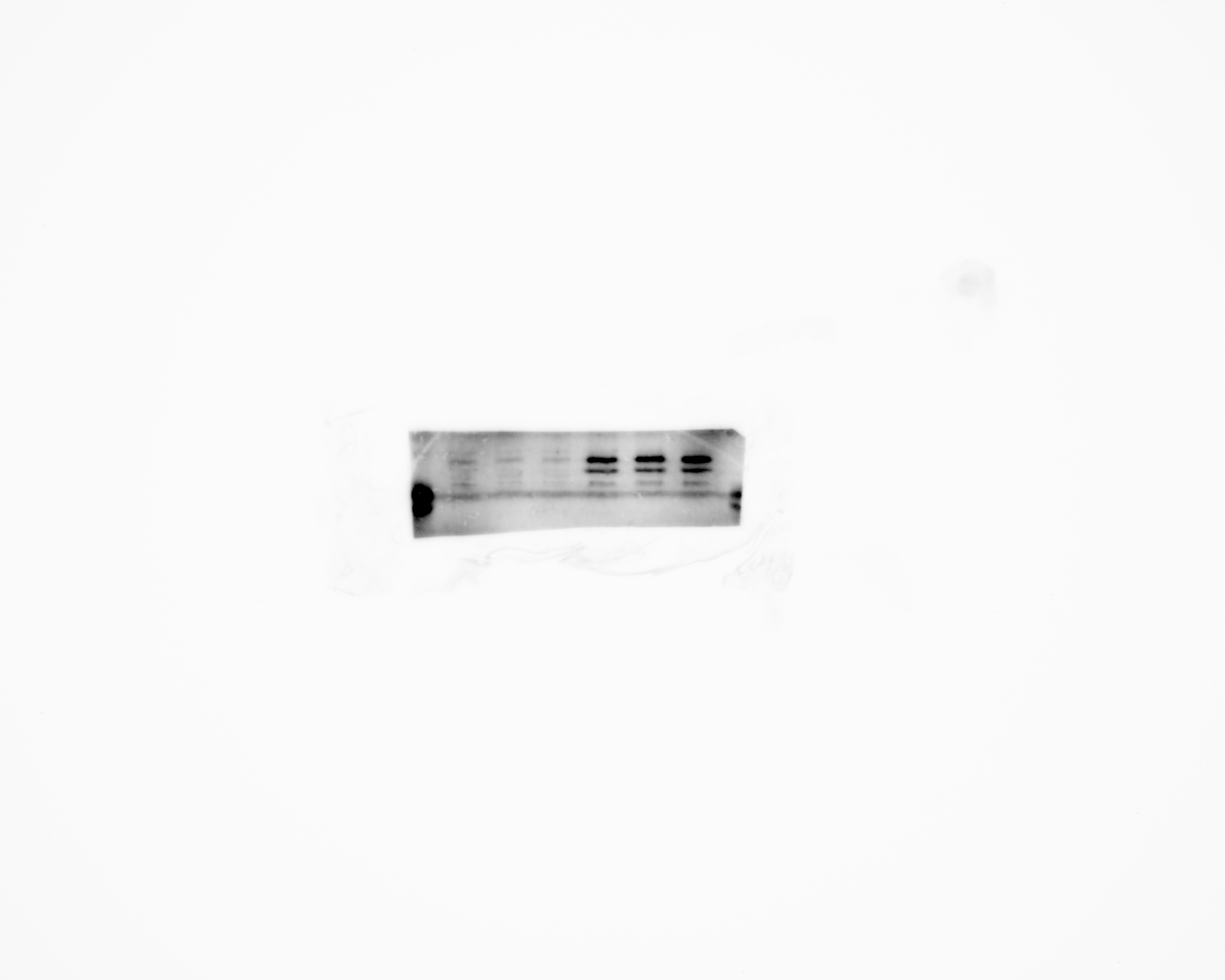

Supplement: Figure 1—figure supplement 3—source data 1. [file elife-100904-fig1-figsupp3-data1.zip › Supplementary Figure 3-source data /Anti-cyclin D1.tif]
